# Human papillomavirus type 16 variants in cervical intraepithelial neoplasia and invasive carcinoma in San Luis Potosí City, Mexico

**DOI:** 10.1186/1750-9378-4-3

**Published:** 2009-02-16

**Authors:** Rubén López-Revilla, Marco A Pineda, Julio Ortiz-Valdez, Mireya Sánchez-Garza, Lina Riego

**Affiliations:** 1 División de Biología Molecular, Instituto Potosino de Investigación Científica y Tecnológica, Camino a la Presa San José 2055, 78216 San Luis Potosí, SLP, Mexico; 2 Clínica de Colposcopía, Jurisdicción Sanitaria 1, Secretaría de Salud de San Luis Potosí, Calzada de Guadalupe 530, 78339 San Luis Potosí, SLP, Mexico; 3 M.C. Marco Antonio Pineda, Área de Ciencias Químico-Biológicas, Universidad del Noreste, Prol. Av. Hidalgo 6315, 89337 Tampico, Tams., Mexico

## Abstract

**Background:**

In San Luis Potosí City cervical infection by human papillomavirus type 16 (HPV16) associated to dysplastic lesions is more prevalent in younger women. In this work HPV16 subtypes and variants associated to low-grade intraepithelial lesions (LSIL), high-grade intraepithelial lesions (HSIL) and invasive cervical cancer (ICC) of 38 women residing in San Luis Potosí City were identified by comparing their E6 open reading frame sequences.

**Results:**

Three European (E) variants (E-P, n = 27; E-T350G, n = 7; E-C188G, n = 2) and one AA-a variant (n = 2) were identified among the 38 HPV16 sequences analyzed. E-P variant sequences contained 23 single nucleotide changes, two of which (A334G, A404T) had not been described before and allowed the phylogenetic separation from the other variants. E-P A334G sequences were the most prevalent (22 cases, 57.9%), followed by the E-P Ref prototype (8 cases, 21.1%) and E-P A404T (1 case, 2.6%) sequences. The HSIL + ICC fraction was 0.21 for the E-P A334G variants and 0.00 for the E-P Ref variants.

**Conclusion:**

We conclude that in the women included in this study the HPV16 E subtype is 19 times more frequent than the AA subtype; that the circulating E variants are E-P (71.1%) > E-T350G (18.4%) > E-C188G (5.3%); that 71.0% of the E-P sequences carry the A334G single nucleotide change and appear to correspond to a HPV16 variant characteristic of San Luis Potosi City more oncogenic than the E-P Ref prototype.

## Background

Human papillomavirus (HPV) types differ from each other by at least 10% of the L1 gene open reading frame (ORF) sequence [1,2]; differences among subtypes are 2%–10%, and less than 2% among variants [1,3-6]. E6 gene sequences can also be used to identify HPV types, subtypes and variants [7].

Persistent infection by high risk-HPV, among which HPV16 is the predominant type, can progress to invasive cervical cancer. The risk of cervical cancer increases with certain HPV16 subtypes [8,9] whose prototype is the European (E-P Ref) subtype [7,10]. Sixty-percent of invasive cervical cancer cases in Mexican women 35 years old or younger are attributed to the Asian-American (AA) HPV16 subtype [11-13], whereas in invasive cervical cancer cases of younger women in Mexico City the AA subtype is 21 times more frequent than the E subtype [14].

Invasive cervical cancer is a public health problem in the state of San Luis Potosí, whose mortality rate in 2005 was above the national average and occupied the tenth place among the 32 federated states [15]. In San Luis Potosí City, the state capital, infection by HPV16 has the highest prevalence [16] and precancerous and cancerous lesions of the cervix are more prevalent in the youngest women (R. López-Revilla and L. Rosales-Ortuño, unpublished data), suggesting that a more oncogenic HPV16 variant may be circulating there.

In this work we identified the HPV16 subtypes and variants in cervical precancerous and cancerous lesions from women residing in San Luis Potosí City by comparing the amplified E6 ORF sequences with those of the HPV16 classes represented in GenBank.

## Results

### Study population

The 38 women included in the study were randomly selected among San Luis Potosí City residents with cervical infection by HPV16 demonstrated by nested PCR amplification of the E6 ORF [16]. Their age range was 22 to 45 years (mean ± SD = 33.2 ± 5.9 years). Twenty seven (71.1%) had low-grade squamous cervical intraepithelial lesions (LSIL), eight (21.1%) had high-grade squamous cervical intraepithelial lesions (HSIL), and three (7.9%) had invasive cervical cancer (ICC).

### Identification of HPV16 subtypes and variants

The 5'-termini of the deposited sequences were aligned to start at nucleotide 83 of the E-P genome, the first one of the ATG start codon of the E6 open reading frame (ORF), and their upstream portions were ignored. Lengths of the deposited sequences ranged from 337 to 576 bp (average = 541 bp). All sequences were longer than the 477 bp expected for the complete E6 ORF, except HPV16-27 (377 bp), HPV16-9 (436 bp), and HPV16-21 (465 bp). The GenBank/EMBL/DDBJ accession numbers of the 38 sequences of this study, EU880235 to EU880272, are depicted in Table 2.

**Table 1:** Primers used

**Pair**	**Primer**	**Sequence (5'→3')**	**Amplicon**
1	LCRS (F)	AAGGGAGTAACCGAAAACGGT	E6-1 (~650 pb)
	
	E7AS (R)	TCATCCTCCTCCTCTGAG	

2	E6F (F)	CGTAACCGAAATCGGTTGAAC	E6-2 (~626 pb)
	
	PU-2R16 (R)	GAGCTGTCGCTTAATTGCTC	

**Table 2:** E6-2 amplicon sequences

	Characteristics of the nucleotide sequences				
	
Cervical sample	Accession number ^a^	Length (bp)	HPV16 subtype	Variant ^b^	Novel change ^a^
HPV16-1	EU880235	521	AA	AA-a	---

HPV16-2	EU880236	573	AA	AA-a	---

HPV16-3	EU880237	537	E	E-T350G	---

HPV16-4	EU880238	560	E	E-T350G	---

HPV16-5	EU880239	539	E	E-T350G	---

HPV16-6	EU880240	576	E	E-T350G	---

HPV16-7	EU880241	562	E	E-T350G	---

HPV16-8	EU880242	493	E	E-T350G	---

HPV16-9	EU880243	436	E	E-T350G	---

HPV16-10	EU880244	539	E	E-C188G	---

HPV16-11	EU880245	545	E	E-C188G	---

HPV16-12	EU880246	562	E	E-P	---

HPV16-13	EU880247	573	E	E-P	A334G

HPV16-14	EU880248	562	E	E-P	A334G

HPV16-15	EU880249	562	E	E-P	A334G

HPV16-16	EU880250	544	E	E-P	A334G

HPV16-17	EU880251	564	E	E-P	A334G

HPV16-18	EU880252	541	E	E-P	A334G

HPV16-19	EU880253	541	E	E-P	---

HPV16-20	EU880254	548	E	E-P	A334G

HPV16-21	EU880255	465	E	E-P	A334G

HPV16-22	EU880256	545	E	E-P	A334G

HPV16-23	EU880257	522	E	E-P	A334G

HPV16-24	EU880258	522	E	E-P	A334G

HPV16-25	EU880259	564	E	E-P	A334G

HPV16-26	EU880260	560	E	E-P	A334G

HPV16-27	EU880261	337	E	E-P	A334G

HPV16-28	EU880262	576	E	E-P	A334G

HPV16-29	EU880263	546	E	E-P	A334G

HPV16-30	EU880264	564	E	E-P	A404T

HPV16-31	EU880265	564	E	E-P	---

HPV16-32	EU880266	563	E	E-P	---

HPV16-33	EU880267	558	E	E-P	A334G

HPV16-34	EU880268	561	E	E-P	A334G

HPV16-35	EU880269	562	E	E-P	A334G

HPV16-36	EU880270	562	E	E-P	A334G

HPV16-37	EU880271	559	E	E-P	A334G

HPV16-38	EU880272	564	E	E-P	A334G

^a ^GenBank/EMBL/DDBJ.

^b ^Nucleotide numbering of the HPV16 E-P Ref genome.

^c ^Described for the first time in this work.

HPV16 subtypes and variants were identified by comparing their sequences with that of the E6 ORF of the HPV16 E-P Ref prototype variant [7]. The HPV16 subtypes identified are listed in Table 2. The predominant one is the European subtype (E) with 36 cases (94.7%); the two cases of the Asian-American (AA) subtype represent 5.3% of the total (Table 2).

**Figure 1:**
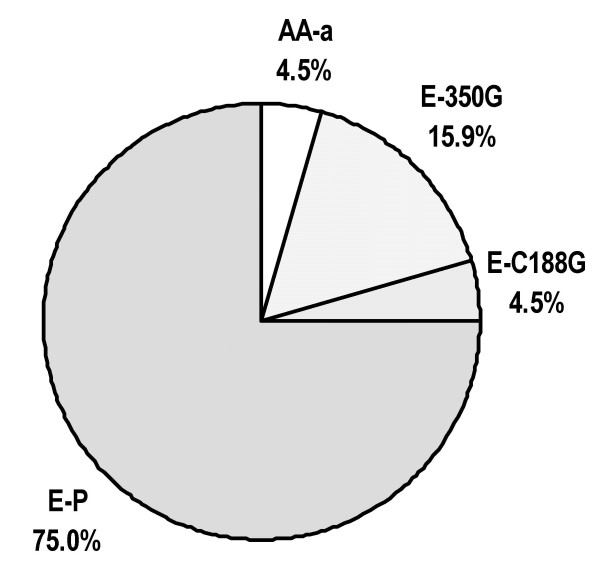
**HPV16 variants identified In San Luis Potosi City**. The sequences of two cases corresponded to the AA-a variant of the Asian-American subtype whereas the 36 remaining cases corresponded to the European subtype (E). Within the E subtype the predominant variant was E-P (n = 27), followed by variants E-T350G (n = 7) and E-C188G (n = 2).

The frequency of HPV16 variants identified is shown in Fig. 1. For the E subtype the most frequent was the E-P Ref variant with 27 cases (71.1%) followed by the E-T350G variant (seven cases, 18.4%) and the E-C188G variant (two cases, 5.3%). AA-a was the only AA variant (two cases, 5.3%).

### Novel HPV16 E6 variants identified

In the E6-HPV16 ORF sequences, 24 single nucleotide changes that had not been described before were found (Table 3); 21 of them (13 substitutions, 6 insertions, 2 deletions) appeared once in nine sequences; the remaining three were observed in two sequences. The A404T change was observed in a single E-P variant.

**Table 3:** Novel polymorphisms identified

HPV16		Nucleotide change		Amino acid change	
Subtype	Variant	Type ^a^	Location ^b^	Substitution	Frameshift

AA	AA-a	D	484	---	127

E	350G	S	A182T	I27L	---
	
	350G	D	393	---	97
	
	350G	S	C206T	Q35stop	---
	
	350G	S	T351A	L83E	---
	
	350G	I	T, 206–207	---	35
	
	G188C	S	A97G	---	---
	
	E-P	S	A404T	I101F	---
	
	E-P	S	C374A	Q91K	---
	
	E-P	D	533	---	144
	
	E-P	I	G, 505–506	---	135
	
	E-P	S	C173A	H24N	---
	
	E-P	S	A330G	---	---
	
	E-P	S	T331A	---	---
	
	E-P	I	TG, 331–332	---	76
	
	E-P	S	A354G	---	---
	
	E-P	S	C360G	---	---
	
	E-P	S	A361G	---	---
	
	E-P	S	A91C	Q-H	---
	
	E-P	I	C, 121–122	---	6
	
	E-P	I	C, 122–123	---	6

^a ^D, deletion. I, insertion. S, substitution.

^b ^Nucleotide numbering of the HPV16 E-P Ref genome.

In 13 sequences there was a deletion of two neighboring bases (AC) located in nucleotide positions 56 and 57, immediately before the ATG protein start codon. The A334G synonymous substitution was found in 22 sequences, located next to nucleotide 335, commonly used for subtype-variant identification.

Amino acid sequences of the E6 oncoprotein encoded by the samples analyzed were compared with the E-P Ref sequence to identify non synonymous mutations (Table 3). E-T350G variants had the expected substitution of leucine for valine at position 83 (L83V). The expected amino acid changes were also found in AA-a (Q14H; H78Y; L83V) and E-C188G variants (E20Q; L83V). Deletion of nucleotide 484, identified in one of the two AA-a variants implies the frameshift in the E6 ORF starting at amino acid 127. The A404T change identified in a single sample, produced the I101F change. The C37A change identified in an E-P variant produced the change Q91K. The C206T change present in an E-T350G variant generated a stop codon instead of amino acid 35 and T351A caused the L83E change in the same sample. The C173A substitution produced the H24N change in one sequence, and the A182T the I27L changes in another one.

### Phylogenetic analysis

**Figure 2:**
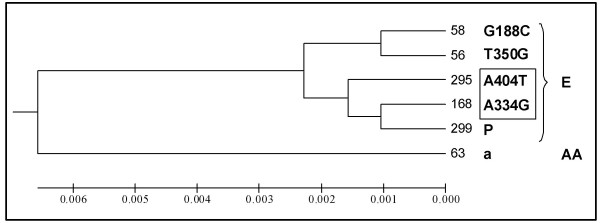
**Phylogenetic tree of the HPV16 variants formed with six representative E6-2 ORF sequences**. Numbers to the right of each branch indicate the identity of the cervical samples; the name of the variant is at the right of the identity number. Capital letters to the extreme right indicate the HPV16 European (E) or Asian-American (AA) subtype to which the variants belong. This optimal phylogenetic tree was constructed to scale, with branch lengths having the same units as the evolutionary distances used to infer them. Note that sequences with the A334G and A404T single nucleotide changes (framed) identified for the first time in this work, as well as the already known C188G, T350G variants are close to the E-P Ref (P) prototype variant.

Identical dendrograms were generated with the 38 E6-2 nucleotide sequences using the Phylip and MEGA programs. Comparing all sequences it was not possible to resolve the A404T non synonymous mutation as a group independent of the E-P Ref sequence. A tree was thus constructed with the six E6-HPV16 ORF sequences representing each of the variants identified, including those containing the single nucleotide changes A334G and A404T. The optimal tree with branch length = 0.01907 was drawn to scale with the same evolutionary distance units used to infer the phylogenetic tree (Fig. 2). In this way the AA-a variant and the three known E-P variants (E-P Ref, E-T350G and E-C188G) could be related, with two new branches of the E subtype corresponding to those carrying the A334G and A404T changes.

### Association of cervical lesions with E-P A334G sequences

The presence of the A334G change in 19 of the 32 E-P sequences (70.4%) makes the corresponding HPV16 "variant" the most prevalent in San Luis Potosi City.

The proportion of HSIL and ICC lesions ("HSIL + ICC fraction") associated to the 19 A334G variants (0.21) was clearly higher than that associated to the eight E-P Ref variants (0.00). Although the HSIL + ICC fraction was even higher for the AA-a, E-T350G and E-C188G variants, the number of samples of these variants were too small to reach definitive conclusions on their oncogenicity (Table 4).

**Table 4:** Cervical lesions associated to the HPV16 variants identified

	Cervical lesions				
		
HPV16 variants	LSIL	HSIL	ICC	Total	HSIL + ICC fraction^a^
E-P Ref	8	0	0	8	0.00

A334G	15	3	1	19	0.21

AA-a	1	1	0	2	0.50

E-T350G	3	4	0	7	0.57

E-C188G	0	1	1	2	1.00

^a ^(HSIL + ICC)/total number of lesions for each variant.

## Discussion

HPV16 is the viral type with the highest association to HSIL and ICC; it was one of the first HPV's to be sequenced [17,18], and it is well known that infection by certain HPV16 subtypes and variants can lead to faster disease progression in younger women [19,20].

HPV subtypes have been identified by comparing the sequences from the E6 and L1 genes and of the long control region (LCR). In the case of HPV16 the E6 gene is frequently used because a short and continuous fragment of its sequence contains sufficient information to identify all the subtypes and variants that have been described [10,21-24]. HPV16 subtypes differ in prevalence, biochemical and biological properties (e.g., replication and expression of AA E6 and E7 oncogenes is more efficient) with uncertain implications in cervical cancer aetiology [12,25-27].

In this work, the first to approach HPV molecular phylogeny in Central Mexico, we identified HPV16 subtypes and variants by comparing the E6 ORF sequences [7]. Through the use of nested PCR enough E6-2 DNA was obtained to sequence the amplified samples. Amplicon length was heterogeneous because the length of the product ends is variable with the sequencing method used [28]. Sequence analysis confirmed that all samples correspond to HPV16, supporting the specificity of the PCR-RFLP method [29] we used for genotyping [16].

Among the HPV16 E6-2 amplicon sequences, 36 corresponded to the E subtype and two to the AA subtype. No African subtypes were identified, as has been the case in previous studies performed in Mexico City [12,14].

Berumen et al. [14] found a 1.1% prevalence of the HPV16 AA subtype in controls and 23.2% in ICC cases in Mexico City, whereas del Refugio Gonzalez-Losa et al. [12] did not find the HPV16 AA subtype. These differences are probably due to the severity of the lesions included since the group of samples analyzed by Berumen et al. [14] had ICC, whereas in this work LSIL predominated.

We identified four HPV16 variants in San Luis Potosi City: E-P (n = 27, 71.1%), E-T350G (n = 7, 18.4%), E-C188G (n = 2, 5.3%) and AA-a (n = 2, 5.3%), whereas Berumen et al. [14] observed frequencies of 47% for the E-T350G and 8% for the E-350T variants. The contrast in the variety of subtypes and variants found by us probably derive also from differences in the kind of lesions as observed above; however, it cannot be ruled out that specific population features can be involved, since we have already found divergence among HPV type frequencies in the neighboring Mexican states of San Luis de Potosí and Guanajuato [16] which also differ from those observed in Mexico City [12,14].

Besides the known point mutations characteristic of the HPV16 variants identified by us, we detected 24 novel single nucleotide changes, two of which appeared in a considerable proportion of the sequences analyzed. The non synonymous A404T substitution, observed only in one sequence close to the E-P Ref, generates the I101F amino acid change in the E6 protein. The second most frequent novel change, observed in 13 cases (34.2%), consists in a deletion of two contiguous bases (AC) in the nucleotide positions 56 and 57 located in the 5'-untranslated region of the E6 gene.

The synonymous A334G, the most frequent of the novel single nucleotide changes found in 22 E subtype sequences (57.9%) is phylogenetically close to the E-P Ref prototype and appear to identify an HPV16 variant characteristic of the region. The E-P A334G variant appears to be more oncogenic, because the proportion of HSIL and ICC lesions were clearly higher for them than with the E-P Ref variants (Table 4). To verify if E-P A334G sequences indeed correspond to a new variant, the complete viral genome must be cloned and sequenced [1].

## Conclusion

HPV16 subtypes and variants infecting the cervix of 38 women from San Luis Potosí City, Mexico, were identified by comparing the sequences of E6 ORF nested PCR products; 36 sequences corresponded to the E subtype and two to the AA subtype.

Three variants of the E subtype were identified: E-P (n = 27, 71.1%), E-T350G (n = 7, 18.4%), E-C188G (n = 2, 5.3%). The only AA variant identified was AA-a (n = 2, 5.3%).

Besides the known point mutations of the E variants identified, 24 novel single nucleotide changes were detected.

The most frequent of the novel changes, found in 19 E subtype sequences, is the synonymous A334G which appears to identify an HPV16 variant characteristic of the region.

The second most frequent novel change, observed in 13 E subtype sequences, is a deletion of two contiguous bases (AC) in nucleotide positions 56 and 57 of the E6 5'-untraslated region.

## Methods

### DNA from cervical scrapings

Randomly selected cervical lesion samples from 38 women residing in San Luis Potosí City, with HPV16 infection diagnosed in our laboratory [16], had been obtained by one of us (JOV) at the Colposcopy Clinic, Secretaría de Salud, San Luis Potosi City.

Each scraping was taken with an endocervical brush ('cytobrush') that was immediately inserted into a 5 mL polypropylene tube (Nalge Nunc, Rochester, NY) containing 1 mL phosphate buffered saline (PBS: 137 mM NaCl, 2.7 mM KCl, 10 mM Na_2_HPO_4_, 2 mM KH_2_HPO_4_, pH 7.4) supplemented with sterile 25 mM disodium ethylene-diaminotetraacetate (EDTA), pH 8.0 (PBS-EDTA). Once detached from the cytobrush and suspended in the PBS-EDTA vehicle, each sample was fixed by addition of 1.5 mL 96% ethanol and processed to extract DNA on the same day or up to 30 days after being kept at room temperature. Reagents were purchased from J.T. Baker (Xalostoc, Mexico) unless other source is specified.

To extract the DNA, each fixed sample was mixed by vortexing and 1 mL transferred to a 1.5 mL tube and spun in a Hettich Mikro 20 microcentrifuge (Cologne, Germany) for 5 min at 13,000 rpm (16,250 × *g*). The supernatant was discarded by decantation and to each pellet were added 500 μL of Tris-EDTA-saline (TES: 10 mM Tris-HCl; 2 mM disodium EDTA, 0.4 M NaCl, pH 8.0 at 25°C), 50 μL of 10% sodium dodecyl sulphate and 20 μL of proteinase K (20 mg/mL). Mixtures were incubated at 55°C for 3 h, at the end of which 150 μL of 5 M NaCl were added and centrifuged again for 15 min. Each supernatant was aspirated and transferred to a tube to which 577 μL cold isopropanol were immediately added and then left stand for 10 min at 4°C to precipitate the nucleic acids. The tubes were centrifuged again for 10 min and the supernatants discarded by decantation. Each pellet was washed by vortexing with 1 mL of 70% cold ethanol and centrifuged for 10 min at 10,000 rpm (9,615 × *g*) and room temperature. Supernatants were discarded by aspiration and the pellets dried out by inverting the tubes for 15 min on a paper towel. Each pellet was dissolved with 50 μL TE (10 mM Tris-HCl, 1 mM disodium EDTA, pH 8.0 at 25°C).

DNA quality was verified by electrophoretic analysis in 1% agarose gels with TAE buffer (40 mM Tris-acetate, 1 mM disodium EDTA, pH 8.2 at 25°C). Two-μL from each sample were applied to gels which were run at 60 V for 90 min. λ-phage DNA digested with *Hind *III (Sigma-Aldrich, Mexico) was used as marker. After staining for 20 min with ethidium bromide (1 μg/mL) gels were transilluminated with ultraviolet light and their fluorescence recorded with the Bio-Rad ChemiDoc EQ (Hercules, CA) photodocumenter.

DNA was quantified by fluorometry with the PicoGreen dsDNA Quantitation kit (Molecular Probes; Eugene, OR) by interpolation in a standard curve containing up to 50 ng of λ-phage DNA. To each well of a black FIA 96 well plate (Greiner Bio-One, Frickenhausen, Germany) 198 μL of the assay solution (PicoGreen diluted 1:400 in TE) and 2 μL of standard DNA or problem samples were added, and their fluorescence determined using a 485 nm excitation filter and a 535 emission filter in the GENios Pro fluorometer (Tecan Systems, San Jose, CA) with the Magellan 4 software.

### Direct and nested PCR

**Figure 3:**
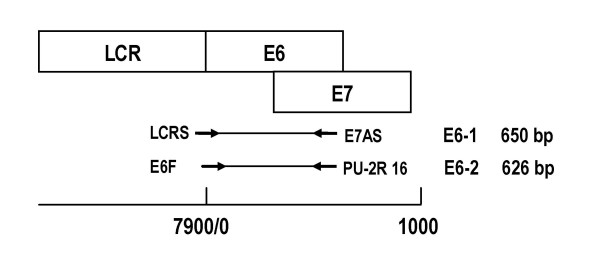
**Direct and nested PCR used to generate E6-1 and E6-2 amplicons**. The upper part of the figure is a diagram of the long control region (LCR) and the E6 and E7 genes, whose sequences partially overlap. The middle part describes the E6-1 amplicon of 650 bp generated by direct PCR with the LCRS/E7AS primer pair and the E6-2 amplicon of 626 bp generated by nested PCR with the E6F/PU-2R 16 primer pair. The lower part is a scale to mark the positions of the LCR, E6 and E7 genes and the E6-1 and E6-2 amplicons in the HPV16 genome.

Nested PCR was used to generate enough DNA to sequence the E6-HPV16 ORF [16]. The E6-1 product (~650 bp) was preamplified with the LCRS/E7AS primer pair in the first reaction (PCR1), and the E6-2 product (~626 bp) with the E6F/PU-2R16 primer pair in the second reaction (PCR2) (Fig. 3, Table 1). PCR1 mixtures of 50 μL contained 2 mM MgCl_2_, the four deoxinucleotide triphosphates (0.4 mM each), forward and reverse primers (0.6 μM each), 1.5 U of *Taq *DNA polymerase and 25 ng of cervical DNA in 200 mM Tris-HCl 500 mM KCl, pH 8.4. Mixtures were preamplified by incubation in a Touchgene Gradient (Techne) thermocycler with initial denaturation at 94°C by 4 min, 40 cycles of amplification (1 min denaturation at 94°C, 1 min annealing at 55°C 1 min extension at 72°C) and 10 min final extension at 72°C. To generate E6-2, to PCR2 mixtures (same composition as PCR1 mixtures except for primers and DNA) 1 μL of each preamplified PCR1 mixture was added as template. Amplification products were electrophoresed in high-resolution sodium borate (SB)1% agarose gels [30].

The intensity of E6-2 bands generated by nested PCR was highest in the presence of 2 mM MgCl2, which was used in all subsequent amplification experiments. In a pilot test E6-2 bands were amplified by direct PCR in positive controls (pHPV16 and pHPV18) and in 23 out of 28 (82.1%) cervical samples. In view of these results we decided to amplify all samples through nested PCR.

**Figure 4:**
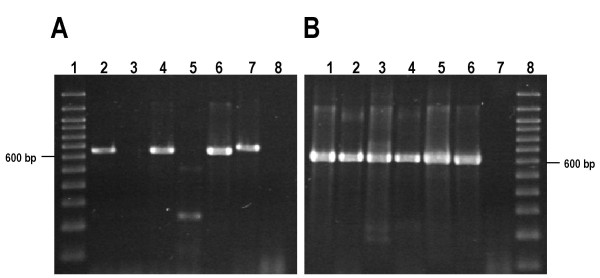
**Nested PCR increases the efficiency of E6 amplicon synthesis**. Examples of amplifications from HPV16-positive cervical scrapings by direct and nested PCR. (**A**) E6-1 amplification from cervical DNA by direct PCR with the LCRS/E7AS primer pair. Lane 1, 100 bp ladder. Lanes 2–5, DNA of cervical samples. Lane 6, Positive control 1 (pHPV16). Lane 7, Positive control 2 (pHPV18). Lane 8, Negative control (water). (**B**) E6-2 amplification by nested PCR with the E6F/PU-2R16 primer pair from direct PCR mixtures. Lanes 1–4, Amplicons generated using direct PCR mixtures from Panel A lanes 2–5 as templates. Lane 5, Positive control 1 (pHPV16). Lane 6, Positive control 2 (pHPV18). Lane 7, Negative control (water). Lane 8, 100 bp ladder.

E6-1 was preamplified in PCR1 mixtures with the LCRS/E7AS primer pair and E6-2 in PCR2 mixtures with the internal E6F/PU-2R16 primer pair. In this way conspicuous E6-2 bands were obtained from all samples with yields sufficient (> 1500 ng) to sequence the amplicons (Fig. 4).

The pHPV16 and pHPV18 plasmids containing the complete genomes of HPV16 and HPV18 respectively, donated by Dr. Alejandro García Carrancá (Instituto Nacional de Cancerología, Mexico City), were used as positive controls. PCR mixtures without DNA were used as negative controls.

### Identification and phylogeny of HPV16 subtypes and variants

The DNA purified with the Wizard kit (PCR Preps DNA Purification Systems, Promega, Madison, WI) from 40 μL of PCR2 mixtures was used to sequence the positive and negative strands of E6-2 amplicons with the method of Sanger et al. [28] at the National Laboratory for Genomic Biodiversity (Guanajuato campus of CINVESTAV, Mexico) and compared with the E6 ORF of the major HPV branches (E, AA, Af1, Af2) by multiple alignment with the ClustalW v1.82 software [31]. Viral subtypes and variants were identified by comparing the E6-HPV16 ORF nucleotide sequences published by Yamada et al. [7]. Allocation of nucleotide and amino acid positions in the E6-HPV16 ORF was based on the nucleotide sequences [32]. Amino acid sequences of the E6 oncoprotein were predicted with the Translate program tool of the ExPASy database [33].

Their evolutionary history was inferred using the UPGMA method [13]. Evolutionary distances were computed using the 2-parameter method [34] whose units are the number of base substitutions per site. Codon positions included were first + second + third + noncoding. All positions containing gaps and missing data were eliminated from the dataset (complete deletion option); there were a total of 477 positions in the final dataset. Phylogenetic analyses were conducted in MEGA4 [35].

## Competing interests

The authors declare that they have no competing interests.

## Authors' contributions

JOV took care of the patients and selected and obtained the cervical samples. MAP performed most of the molecular studies and the bioinformatics analyses. MSG collaborated in PRC amplification experiments and sequencing. LR directed and supervised the bioinformatics analyses. RLR conceived and designed the study, obtained the funds to carry it out and drafted the manuscript. All authors read and approved the final manuscript.
